# Web-Based, Computer-Tailored, Pedometer-Based Physical Activity Advice: Development, Dissemination Through General Practice, Acceptability, and Preliminary Efficacy in a Randomized Controlled Trial

**DOI:** 10.2196/jmir.1959

**Published:** 2012-04-24

**Authors:** Katrien De Cocker, Heleen Spittaels, Greet Cardon, Ilse De Bourdeaudhuij, Corneel Vandelanotte

**Affiliations:** ^1^Department of Movement and Sports SciencesGhent UniversityGhentBelgium; ^2^Centre for Physical Activity StudiesInstitute for Health and Social Science ResearchCentral Queensland UniversityRockhamptonAustralia

**Keywords:** Physical activity intervention, step counts, computer tailoring

## Abstract

**Background:**

Computer tailoring is a relatively innovative and promising physical activity intervention approach. However, few computer-tailored physical activity interventions in adults have provided feedback based on pedometer use.

**Objectives:**

To (1) describe the development of a Web-based, pedometer-based, computer-tailored step advice intervention, (2) report on the dissemination of this tool through general practice, (3) report on its perceived acceptability, and (4) evaluate the preliminary efficacy of this tool in comparison with a standard intervention.

**Methods:**

We recruited 92 participants through general practitioners and randomly assigned them to a standard condition (receiving a pedometer-only intervention, n = 47) and a tailored condition (receiving a pedometer plus newly developed, automated, computer-tailored step advice intervention, n = 45). Step counts, self-reported data obtained via telephone interview on physical activity, time spent sitting, and body mass index were assessed at baseline and postintervention. The present sample was mostly female (54/92, 59%), highly educated (59/92, 64%), employed (65/92, 71%), and in good health (62/92, 67%).

**Results:**

Recruitment through general practitioners was poor (n = 107, initial response rate 107/1737, 6.2%); however, the majority of participants (50/69, 73%) believed it is useful that general practitioners help patients find ways to increase physical activity. In the tailored condition, 30/43 (70%) participants requested the computer-tailored step advice and the majority found it understandable (21/21, 100%), credible (17/18, 94%), relevant (15/18, 83%), not too long (13/18, 72%), instructive (13/18, 72%), and encouraging to increase steps (16/24, 67%). Daily step counts increased from baseline (mean 9237, SD 3749 steps/day) to postintervention (mean 11,876, SD 4574 steps/day) in the total sample (change of 2639, 95% confidence interval 105–5172; *F*_1 _= 5.0, *P *= .04). No interaction or other time effects were found.

**Conclusions:**

The majority of participants in the tailored condition accepted the step advice and indicated it was useful. However, in this selected sample of adults, the tailored condition did not show superior effects compared with the standard condition.

## Introduction

There is ample evidence of the positive effects of regular physical activity on physical and mental health [1]. International guidelines recommend at least 150 minutes/week of moderate to vigorous physical activity to prevent chronic diseases, such as cardiovascular diseases, type 2 diabetes mellitus, obesity, hypertension, and some cancers [2,3]. As most adults are not meeting this recommendation [4-6], several interventions to promote physical activity have been developed and implemented [7,8].

A relatively innovative and promising intervention approach in public health promotion is the use of computer tailoring via the Internet [9,10]. This technique mimics and automates the process of individual counseling, by giving participants immediate personally adapted feedback about their assessed behavior. After the completion of an online diagnostic questionnaire, the computer automatically translates this information, through a series of “if–then” statements, into individualized feedback [11]. This tailoring of messages is found to be a more effective technique to guide behavior change than is providing generic or standard advice [10]. However, several authors advocate more research on computer-tailored health programs [9,10].

The major advantage of computer tailoring through the Internet is the ability to reach many people in a variety of settings at any time and location, and at a relatively low cost. Research revealed that promoting physical activity via the Internet is feasible and appealing to adults [12,13]. Furthermore, the Internet now has more than one billion users worldwide [14] and, as such, has become a mainstream intervention-delivery channel. In Europe, 60% of the population uses the Internet daily [15].

Existing computer-tailored physical activity programs might, however, also have weaknesses. As the diagnostic assessment is mostly done by questionnaires [9], the self-reported data may have recall biases [16]. Consequently, using more objective outcome measures [11] might be a more appropriate way of assessing baseline physical activity levels in the process of providing computer-tailored feedback. The most commonly used, more objective way of assessing physical activity is measuring step counts through the use of step counters or pedometers [17]. Pedometers have become popular monitoring tools for physical activity in large free-living populations [17]. However, to our knowledge, few computer-tailored interventions in adults have provided feedback based on pedometer use [18,19]. Nevertheless, some potential benefits are associated with integrating pedometers in computer-tailored physical activity feedback. First, the accuracy of assessing the targeted behavior will increase, as it would be based on more objectively measured step counts. Second, the computer-tailored advice could specifically target step count increases through pedometer use and, as such, the pedometer would be used not only as an assessment instrument but also as an intervention tool. Due to the self-monitoring aspect of and the ongoing feedback provided by the pedometer, resulting in increased awareness and motivation, the device can be used as a behavior modification tool. In addition, goal setting, a behavioral change strategy used in many computer-tailored programs [9,10,20,21], can easily be facilitated through pedometer use [22]. Pedometer-based behavioral modification programs have already shown positive effects [23,24]; however, providing continuous face-to-face feedback is time consuming and expensive. Offering an additional online, computer-tailored tool in pedometer-based interventions might thus be beneficial.

Another weakness of online physical activity programs is reaching the targeted population. Recruiting individuals to visit website programs on health behavior change appears to be rather difficult. For example, Australian research conducted in a worksite sample showed that only 46% of participants who agreed to take part in a website-delivered physical activity intervention actually visited the website [25]. A computer-tailored program wherein computers and printers were installed in waiting rooms of general practices in Rhode Island also showed low rates (0%–12%) of use [26]. However, a Belgian study found that brief face-to-face contact when handing out flyers increased recruitment rates (46%) when compared with recruiting via flyers only (6%) [20]. As such, a possible promising dissemination channel of online physical activity interventions is the visit to primary health care [26], as general practitioners (GPs) have personal face-to-face contact with their patients but often lack the time or the skills to provide counseling on health behaviors themselves [27,28]. In addition, GPs are perceived as a highly credible source of influence concerning health aspects [29]. The objectives of the present paper were to (1) describe the development of an online pedometer-based, computer-tailored physical activity step advice intervention, (2) report on the dissemination of this tool through general practice, (3) report on its perceived acceptability among participants recruited through GPs, and (4) evaluate the preliminary efficacy of the new intervention in comparison with a standard pedometer-only intervention.

## Methods

### Development of the Pedometer-Based, Computer-Tailored Physical Activity Advice

The development of computer-tailored interventions requires (1) a data source, including the significant characteristics of the recipient derived from an individual diagnosis or assessment, (2) a message library that contains the intervention messages, (3) a set of decision rules that selects messages matched and tailored to the specific needs of the recipient, and (4) a channel that delivers the messages to the specific person, such as the Internet [30]. Based on previous computer-tailored interventions to increase physical activity in a Flemish population [31-34], a computer-tailored step advice intervention was developed. In this Web-based intervention, participants received personalized feedback on the amount of steps they take daily, and were provided with tips and suggestions on how they can take more steps if needed. The general approach, structure, and theoretical background of the new computer-tailored step advice intervention remained the same as in the previous developed computer-tailored interventions; however, the focus was changed from increasing physical activity to increasing steps. As such, the 10,000 steps concept [35,36] was integrated into the present intervention. Multimedia Appendix 1 presents some examples of screenshots of the automated Web-based advice intervention.

Prior to visiting the computer-tailored website, participants’ baseline step level had to be determined. Participants were instructed to wear a pedometer for 7 consecutive days without changing their usual lifestyle. To receive the computer-tailored step advice, participants had to log on to a website using a confidential username and password, and then complete a questionnaire (see Multimedia Appendix 1, Figure A and B). This questionnaire assessed participant’s demographics, baseline step level, and the psychosocial correlates of achieving 10,000 steps/day. As soon as participants had completed all the questions, tailored feedback was provided on the computer screen.

The tailored feedback was created from a database of messages that match any possible combination of answers and is based on the theory of planned behavior [37] and the transtheoretical model [38]. The theory of planned behavior was considered by giving feedback about participants’ intentions, attitudes, self-efficacy, social support, knowledge, benefits, and barriers related to physical activity. We considered the stages of change in two ways. First, the content differed between stages. Precontemplators mainly received general information about the 10,000 steps concept and about its health benefits. To avoid resistance, the need for behavior change was not dictated, but only vaguely suggested. Contemplators received the same information, although not so extensively, and it was mentioned that they might benefit from taking more steps. In the preparation stage, the emphasis was on increasing steps, combined with specific step and health information. In the action stage, the emphasis was on keeping up the steps and preventing relapse. In the maintenance stage, feedback was reduced to saying that they were doing well and that they should carry on. Second, the way in which the participants were approached also differed between stages. Information for precontemplators was presented in an impersonal way (eg, people could...), again avoiding resistance. Contemplators were approached in a personal way (eg, you could...), but not in the decisive way that was used for preparators (eg, you should...) or the supportive way used for people in the action or maintenance phase (eg, you do...).

The feedback was organized so that participants first received a general introduction (see Multimedia Appendix 1, Figure C), followed by normative feedback, which relates participants’ step level to the goal of 10,000 steps/day. Based on baseline step levels, a schedule was provided on how they could reach the goal of 10,000 steps/day over time (participants could choose to increase their current steps by 500 or 1000 per week [39]; see Multimedia Appendix 1, Figure D). Progress feedback (positive or negative evolution) was provided when participants requested advice for a second time or more; it compared the previous step level with the current level. Next, participants received tips on how to increase steps (if needed) during work, household chores, gardening, leisure time, and transport (see Multimedia Appendix 1, Figure E). This further included information on what a walking buddy is, how step guidelines compare with overall physical activity guidelines, how to correctly use a pedometer, what benefits originate from 10,000 steps/day, how to deal with barriers associated with stepping more, how to overcome low self-efficacy to take more steps, and how the local environment can provide opportunities to walk. Altogether the feedback, which can be printed, could amount to as much as 5 or 6 pages of advice. Table 1 provides some examples of the introduction to the tips and suggestions part of the advice intervention for the various stages of change.

**Table 1:** Examples of introductions to the tips and suggestions of the advice intervention for the various stages of change.

Stage of change	Example of introduction to the tips and suggestions section of the pedometer-based, computer-tailored advice
Precontemplation	It seems that you are not reaching the goal of 10,000 steps a day. That’s a pity because being active has several health advantages, in both the short and the long term. People can experience these benefits when they are being physically active on a regular basis. The following tips could help people who want to be more active...
Contemplation	It seems that you are not reaching the goal of 10,000 steps a day, but are planning to become more active at some point in the future. That’s good because being active has several health advantages, in both the short and the long term. You could experience these benefits when you are being physically active on a regular basis. When you decide the time has come to take more steps, the following tips and suggestions will certainly be helpful...
Preparation	You are intending to take more steps than you are taking now, and you want to reach this goal within 1 month. This is a good idea, as you are currently not reaching the goal of 10,000 steps a day which is needed to achieve health benefits. The following tips should help you to realize your good intentions...
Action	Because you are already reaching the 10,000 steps goal, it doesn’t seem necessary to overload you with tips to take even more steps. After all, you are doing well! Still, we want to give you some tips, which may be helpful in times when it is hard to keep up your high level of physical activity...
Maintenance	Because your step level is high and you have been able to maintain this for quite a while, it seems unnecessary to give you tips to step more. They would probably not be very helpful. However, we want to emphasize that you are among the few Flemish people who are very active, and that’s really good! Carry on being this active!

### Pretesting Procedures of the Computer-Tailored Step Advice

Through contacts with GPs’ organizations, we found a convenience sample of 38 GPs willing to take part in this pretest study to evaluate the dissemination and test the acceptability of the computer-tailored step advice. We recruited participants through GPs, who were asked to personally hand out invitation letters to the 50 first counseling patients eligible for the study. Exclusion criteria were (1) being physically unable to engage in physical activity, (2) already being highly active or participating in sport activities, and (3) not being Dutch speaking. The letter briefly explained the purpose of the project, namely promoting physical activity in the general population through pedometer use and providing computer-tailored feedback; invited the patient to take part in the study; and presented the inclusion criteria: (1) being aged 18–65 years, (2) having Internet access at home or at work, and (3) having a personal email address. To participate, they were required to email us their full name, address, telephone number, date of birth, and name of GP.

On receiving this information from the participants, we sent them an envelope containing a pedometer, a step log for 7 days, information on how to use these instruments, and a stamped, self-addressed envelope for return mailing after 3 months. Participants were first asked to wear the pedometer for 7 consecutive days and to complete the step log in order to assess their baseline step level; they were asked not to increase their step or activity levels from what they would usually do in this period. Afterward, they had to email the step log to us. After receiving the step log, we contacted the participants by telephone to complete the baseline assessment.

After this interview, we randomly assigned participants to (1) the pedometer intervention only (standard condition) or (2) the pedometer intervention supplemented with computer-tailored step advice (tailored condition). Participants in both conditions were mailed generic paper booklets with information on how to increase their steps [35,36]. Participants in the tailored condition also received a login and password to enter the website that provided the computer-tailored step advice. Every month, we checked whether participants had requested the computer-tailored step advice. If they did, we emailed them an invitation to access the computer-tailored step advice for a second or third time to receive feedback on their progress. If they did not, a reminder was emailed to reinvite participants to request the computer-tailored step advice for the first time. At 3 months, we asked participants to report a second step count registration of 7 consecutive days and to participate in a second telephone interview.

Participants completed informed consent forms, and the study protocols were approved by the Ethics Committee of the Ghent University, Belgium. The study was conducted between January and August 2010.

### Measures

#### Pedometer

We used the Yamax Digiwalker SW-200 (Yamax, Tokyo, Japan) in this study, as it is known to be a valid, accurate, and reliable instrument for counting steps in adults [40].

#### Step Log

Participants were requested to record the date, the daily steps taken, and the type and duration of nonambulatory activities (eg, biking and swimming) in an activity log. Following established guidelines [41], we added 150 steps to the daily total for every minute of reported biking or swimming.

#### Demographics

During a telephone interview at baseline and postintervention, we asked participants their gender, age, height, and weight. The interviewers also obtained information on participants’ perceived health (very good, good, moderate, poor, or very poor), education (primary education, vocational secondary education,; technical secondary education, general secondary education, and college or university), employment status (yes or no), computer and Internet use (daily, weekly, monthly, a couple of times a year, or never), and Internet access at home or at work (yes or no). Furthermore, participants were asked who gave them the invitation letter (GP or other; in the case of answering other, participants were asked to specify). Finally, we assessed their intention to participate in physical activity by asking whether participants planned to increase their steps (yes, within 1 month; yes, within 6 months; or no intention).

#### International Physical Activity Questionnaire

To assess physical activity and time spent sitting, we used the long interview form of the International Physical Activity Questionnaire (IPAQ) at baseline and postintervention. Physical activity in a usual week in four different domains was measured: at work, during transport, at home, and during leisure time. The IPAQ has been shown to be a valid and reliable instrument at the population level in Europe [42] and in Flanders, Belgium [43].

#### Feasibility Telephone Interview

At postintervention, we assessed the feasibility of disseminating the intervention through GPs. We asked all participants about the usefulness of GPs emphasizing the importance of sufficient physical activity, helping to find ways to increase steps, and providing pedometers to their patients, using 5-point Likert scales (ranging from totally not useful to very useful).

#### Acceptability Telephone Interview

At postintervention, we asked participants in the tailored condition about the understandability, logic, practical use, and length of the questionnaire prior to receiving the advice. Four questions assessed what participants did with the advice (read it, discussed it with others, saved it, or reread it later). The interviewer also asked what the advice indicated about the step level of participants (insufficient, just enough, or sufficient) and whether participants were aware of this. Further, participants were asked about the relevance, credibility, understandability, and length of the advice; whether the advice helped them to gain insight into their physical activity pattern; and whether the advice was an encouragement to increase steps. If participants requested the advice more than once, they were asked about the usefulness of receiving the advice twice or more.

### Data Analyses

We analyzed all data using SPSS 17.0 for Windows (IBM Corporation, Somers, NY, USA). The level of statistical significance was set at .05. Participant characteristics were described using descriptive statistics. Self-reported physical activity was expressed in minutes/day for total time spent walking and total physical activity, and in hours/day for sitting time (based on guidelines at www.ipaq.ki.se). Walking and total physical activity scores were log transformed to obtain normal distributions. However, for clarity, the numbers in the tables are the means and standard deviations of the nontransformed data. Average daily step counts were calculated, and values over 20,000 steps/day were truncated as 20,000 to limit unrealistically high averages and to ensure normal distributions [44]. All participants provided at least 5 days of pedometer registration.

We compared participant characteristics at baseline between the two conditions using independent-samples *t *tests (quantitative variables) and chi-square tests (qualitative variables). The same tests were used to compare baseline characteristics between participants who dropped out and those who did not. Descriptive statistics (numbers, percentages) were used to report on the feasibility of disseminating the intervention through GPs and to test the acceptability of the computer-tailored step advice. Participants who requested the computer-tailored step advice and those who did not were compared using independent-samples *t *tests (quantitative variables) and chi-square tests (qualitative variables).

The time and intervention effects on body mass index (BMI), self-reported and pedometer-based physical activity, and sitting time were examined using repeated measures analyses of variance with condition as the between-participants factor and time as the within-participants factor. We conducted these analyses using both a retained-sample analysis (only participants who completed postintervention assessments) and an intent-to-treat analysis (assuming baseline values at postintervention for dropout participants). As we found no differences between these two types of analyses, we report only the results of the retained-sample analysis.

## Results

### Study Sample

Most of the sample were female (54/92, 59%), were highly educated (59/92, 64%), were employed (65/92, 71%), were in good health (62/92, 67%), used the computer (75/91, 82%) and the Internet (69/92, 75%) daily, and did not reach 10,000 steps/day (65/87, 75%) at baseline. Table 2 presents characteristics of the participants in both conditions. No significant differences were found between the two conditions at baseline for the demographic variables, use (and access) of PC and Internet, intention to change physical activity, self-reported and pedometer-based physical activity, and time spent sitting (see Table 2).

**Table 2:** Participant characteristics at baseline.

Characteristic		Tailored condition (n = 45)	Standard condition (n = 47)	Group comparison	*P *value
**Demographic variable**					
	Age (years), mean (SD)	46.6 (10.9)	47.7 (11.4)	*t*_90 _= 0.5	.63
	Male, n (%)	17/45 (38%)	21/47 (45%)	χ^2^_1 _= 0.5	.50
	BMI^a ^(kg/m^2^), mean (SD)	25.8 (4.3)	26.3 (4.6)	*t*_76 _= 0.5	.64
	Higher education, n (%)	30/45 (67%)	29/47 (62%)	χ^2^_1 _= 0.2	.62
	Employed, n (%)	35/45 (78%)	30/47 (64%)	χ^2^_1 _= 2.2	.14
	Good to very good health, n (%)	26/45 (58%)	36/47 (77%)	χ^2^_1 _= 3.7	.06
**Computer/Internet use and Internet access, n (%)**					
	Daily computer use	36/45 (80%)	39/46 (85%)	χ^2^_1 _= 0.4	.55
	Daily Internet use	30/45 (67%)	39/47 (83%)	χ^2^_1 _= 3.3	.07
	Internet access at home	44/45 (98%)	46/46 (100%)	χ^2^_1 _= 1.0	.31
	Internet access at work	28/35 (80%)	22/30 (73%)	χ^2^_1 _= 3.4	.19
**Intention to change physical activity, n (%)**				χ^2^_2 _= 2.3	.31
	Within 1 month	23/43 (54%)	19/42 (45%)		
	Within 6 months	11/43 (26%)	8/42 (19%)		
	No intention	9/43 (21%)	15/42 (36%)		
**Self-reported physical activity (minutes/day), mean (SD)**					
	Walking	33.2 (60.3)	44.9 (57.4)	*t*_50_= 0.8	.43
	Total physical activity	142.7 (123.8)	163.6 (120.8)	*t*_65 _= 1.7	.10
Pedometer-based physical activity (steps/day), mean (SD)		8609 (3370)	8933 (3367)	*t*_85 _= 0.4	.66
Sitting time (hours/day), mean (SD)		7.0 (3.1)	7.0 (3.4)	*t*_87 _= 0.0	.97

^a ^Body mass index.

In total, 23 participants dropped out: 7 had health problems, 3 lacked the time, and 1 went abroad. The other 12 dropout participants could not be reached at postintervention, so the reason for dropout is unknown. Dropout analyses revealed no significant differences between those who dropped out (n = 13 in the tailored condition; n = 10 in the standard condition) and those who did not (data not shown).

### Feasibility of Dissemination Through GPs

**Figure 1:**
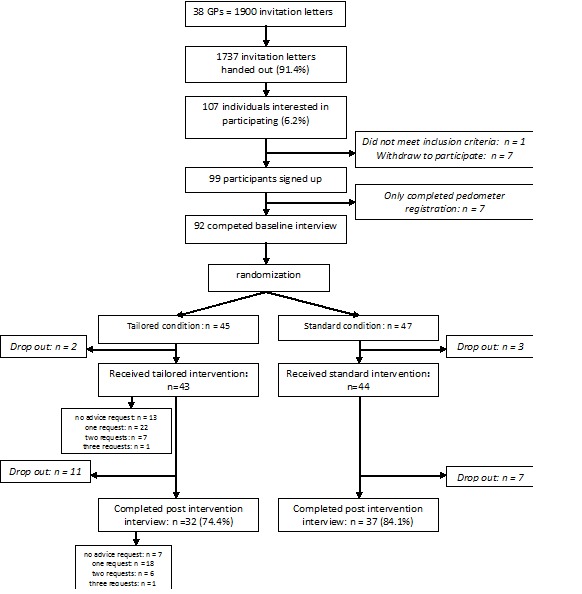
Flow of participants through the study. GP = general practitioner.

From the 1900 available invitation letters (50 per GP, 38 GPs), 1737 letters were handed out to patients. A total of 107 individuals expressed an interest in participating (response rate 6.2%); however, 1 participant did not meet the inclusion criteria and 7 eventually withdrew for family- or work-related reasons, leaving 99 participants at baseline (see Figure 1). The baseline interview (completed by 92 participants) showed that 89 participants had received the invitation letter from their GP, 1 person found it in the waiting room, 1 received it from his wife, and 1 received it from a parent. At postintervention (interview completed by 69 participants), the majority believed that it is useful to very useful that GPs emphasize the importance of sufficient physical activity (61/69, 88%), that it is useful to very useful for GPs to offer pedometers to their patients (61/69, 88%), and that it is useful to very useful for GPs to help patients find ways to increase physical activity (50/69, 73%).

### Acceptability of Web-Based, Computer-Tailored Step Advice

Figure 1 shows that 30% (n = 13) of participants in the tailored condition did not request the computer-tailored step advice, while the other 70% (n = 30) did request the advice at least once: 22 requested it once (51%), 7 twice (16%), and 1 three times (2%). Characteristics of these individuals are presented in Table 3. At baseline, those who requested the advice took fewer steps (*P *= .03) and used their computer less on a daily basis (*P *= .04) than those who did not request the advice (see Table 3). No other characteristics differed significantly between those who requested the advice and those who did not.

Of the 43 participants who received the tailored intervention, 32 completed the postintervention telephone interview (74%). Of this group, 7 did not request the computer-tailored step advice (22%), 18 requested it once (56%), 6 twice (19%) and 1 person three times (3%). The most frequently mentioned reason for not requesting the advice was lack of time; 1 person had computer problems; and 1 believed that he didn’t need the advice. Of those who did request the advice, all found the questions prior to receiving the advice understandable (21/21, 100%), and most had no problems answering them (24/25, 96%), found the questions logically built up (19/20, 95%), and didn’t find the questionnaire too long (14/19, 74%).

After receiving the advice, almost everyone read it (20/21, 95%) and the majority saved it (12/20, 60%). Fewer participants discussed it with others (8/19, 42%), printed it (8/21, 38%), or reread it later (7/20, 35%). Of those who could remember the feedback on their step level, almost half (9/19, 47%) got the advice that they were insufficiently active. The majority (13/19, 68%) had expected the feedback they got. Of those requesting the advice more than once (n = 7), all found it useful to be able to receive the advice several times.

Everyone found the advice understandable (100%), and the majority found the advice credible (17/18, 94%), relevant (15/18, 83%), and not too long (13/18, 72%). The majority also reported that the advice helped them to gain insight into their physical activity pattern (13/18, 72%), and two-thirds found that the advice encouraged them to increase their number of steps (16/24, 67%).

**Table 3:** Baseline characteristics of the participants in the tailored condition who requested the computer-tailored step advice at least once and those who did not request the computer-tailored step advice.

Characteristic		At least one request (n = 30)	No request (n = 13)	Group comparison	*P *value
**Demographic variable**					
	Age (years), mean (SD)	47.2 (11.2)	43.5 (9.9)	*t*_26 _= 1.0	.31
	Male, n (%)	11/30 (37%)	4/13 (31%)	χ^2^_1 _= 0.1	.76
	BMI^a ^(kg/m^2^), mean (SD)	26.1 (4.7)	24.4 (2.7)	*t*_20 _= 1.0	.32
	Higher education, n (%)	19/30 (63%)	9/13 (69%)	χ^2^_1 _= 0.1	.71
	Employed, n (%)	25/30 (83%)	10/13 (77%)	χ^2^_1 _= 0.2	.62
	Good to very good health, n (%)	16/30 (53%)	9/13 (69%)	χ^2^_1 _= 0.9	.33
**Intention to change physical activity, n (%)**				χ^2^_2 _= 2.2	.34
	Within 1 month	13/29 (45%)	9/13 (69%)		
	Within 6 months	9/29 (31%)	2/13 (15%)		
	No intention	7/29 (24%)	2/13 (15%)		
**Computer/Internet use and Internet access, n (%)**					
	Daily computer use	22/30 (73%)	13/13 (100%)	χ^2^_1 _= 4.3	.04^b^
	Daily Internet use	18/30 (60%)	11/13 (85%)	χ^2^_1 _= 2.5	.11
	Internet access at home	30/30 (100%)	12/13 (92%)	χ^2^_1 _= 2.4	.12
	Internet access at work	19/25 (76%)	9/10 (90%)	χ^2^_1 _= 1.0	.61
**Self-reported physical activity (minutes/day), mean (SD)**					
	Walking	30.1 (43.4)	41.0 (66.2)	*t*_14 _= 0.0	.99
	Total physical activity	142.7 (128.0)	153.9 (124.9)	*t*_40 _= 1.1	.28
Pedometer-based physical activity (steps/day), mean (SD)		7690 (2416)	10,730 (4319)	*t*_15 _= 2.4	.03^b^
Sitting time (hours/day), mean (SD)		7.0 (3.2)	7.3 (2.9)	*t*_26 _= 0.3	.77

^a ^Body mass index.

^b ^.01 < *P *< .05.

### Preliminary Efficacy

Table 4 presents the intervention and time effects on self-reported and pedometer-based physical activity, BMI, and sitting time. Daily step counts increased significantly from baseline (mean 9237, SD 3749) to postintervention (mean 11,876, SD 4574) in both conditions (change of 2639, 95% confidence interval 105–5172; *P *= .04); no other significant time or intervention effects were found (see Table 4). In the tailored condition only, no intervention or time effects were found for those who did request the advice and those who did not (data not shown).

**Table 4:** Effects on body mass index, physical activity, and time spent sitting in both conditions.

Variable/condition			n	Baseline	Postintervention	Change (95% CI^a^)	*F*_1 _(time × condition)	*P *value	*F*_1 _(time)	*P *value
**Body mass index (kg/m^2^), mean (SD)**							0.7	.40	3.5	.07
	Tailored condition		27	26.3 (4.7)	26.0 (4.5)	–0.3 (–0.6 to 0.0)				
	Standard condition		33	26.2 (4.6)	26.0 (4.6)	–0.2 (–0.5 to 0.3)				
**Self-reported physical activity (minutes/day), mean (SD)**										
	Walking						0.1	.82	0.1	.71
		Tailored condition	21	17.8 (21.8)	26.4 (34.6)	8.6 (–6.6 to 23.9)				
		Standard condition	22	46.2 (59.7)	38.9 (44.8)	–7.3 (–40.8 to 26.1)				
	Total physical activity						2.0	.16	0.5	.47
		Tailored condition	32	131.0 (121.1)	142.0 (108.9)	11.0 (–25.5 to 47.5)				
		Standard condition	36	165.9 (125.7)	138.2 (98.5)	–27.8 (–72.5 to 16.9)				
**Pedometer-based physical activity (steps/day), mean (SD)**							1.1	.31	5.0	.04^b^
	Tailored condition		10	9162 (2542)	10,668 (3826)	1505 (–1850 to 4861)				
	Standard condition		10	9549 (4903)	13,690 (4743)	4141 (462 to 8744)				
**Sitting time (hours/day)**							0.0	.85	2.4	.12
	Tailored condition		28	7.1 (3.0)	6.6 (3.1)	–0.5 (–1.5 to 0.4)				
	Standard condition		35	7.1 (3.6)	6.4 (3.2)	–0.7 (–1.9 to 0.5)				

^a ^Confidence interval.

^b^ *P *< .05

## Discussion

We developed a new pedometer-based, computer-tailored step advice intervention and examined the feasibility of disseminating this tool through general practice, and its acceptability and preliminary efficacy in adults. Overall, participants accepted the computer-tailored step advice well. Results are comparable with the previous computer-tailored advice intervention [33] on which this pedometer-based, computer-tailored advice intervention was based. Nearly the same number of participants (95% here vs 96%–100% in the original study) read the advice, while even more participants in the present study (94% vs 57%–66%) rated the advice as credible. This might be because we invited participants via GPs, who are perceived as a well-established authority on health issues [29], or because we based the advice on objectively measured step counts. On the other hand, fewer participants discussed the advice with others (42%) and printed it (38%), compared with the previous study (59%–64% and 55%–75%, respectively) [33]. In the Australian study of Leslie et al, only 10% of participants printed out any information from the website physical activity program [25].

Despite this positive evaluation of the computer-tailored step advice, the tool did not result in significant effects on behavior or BMI, compared with participants who did not receive the advice. The evidence on this matter is inconsistent: while some physical activity programs did find good outcomes [34,45], others did not [46,47]. None of these studies, however, were pedometer based. We found only one study [48] that used a personal activity monitor, a uniaxial accelerometer, in combination with tailored physical activity advice through the Internet in the general population (adolescents aged 12–18 years and young adults aged 25–35 years). In the adolescent group, findings suggested promising intervention effects on moderate-intensity physical activity among girls and sedentary time among boys [49]. The intervention in the young working population appeared to be easily applicable to real-life settings, but it was ineffective in improving physical activity behavior or its determinants in healthy office workers [19]. The efficacy of the personal activity monitor study in office workers seems comparable with the present findings; however, in the study of Slootmaker et al only 39% of the users found the advice appealing, while in the present intervention this was evaluated more positively [19].

The fact that the standard condition in the present study was not a true nonintervention may partially explain the lack of interaction effects. Participants in our standard condition used a pedometer and received standard 10,000 steps intervention materials, two strategies that have been shown to be effective in increasing physical activity in adults [36]. A lack of statistical power likely also affected the current outcomes. A priori power analysis indicated that 23 individuals in each intervention condition (total n = 46) would have to participate to achieve sufficient power. This was the case for most self-reported data, but unfortunately only 20 did actually provide objective step count data on both baseline and postintervention measurements. It should also be noted that, even though the majority of the sample did not reach 10,000 steps/day at baseline, most individuals (68%) were already somewhat active (at least 7500 steps/day), which could also explain the lack of effects.

The initial response rate to the invitation letter spread by GPs was very poor. Only 6% signed up for the project after receiving an invitation letter from their GP; we did, however, expect that more participants would respond, as previous research showed that face-to-face contact significantly increased recruitment for an online tailored intervention (46%) compared with recruitment via a flyer only (6%) [20]. A possible explanation for the low recruitment rate might be that some GPs simply put the invitation letters in their waiting room or did not provide any explanation about the study when handing out the invitation letters to their patients. Apart from 1 participant indicating having found the leaflet in a GP’s waiting room, no further details are available on how GPs delivered the invitation letters. Australian research showed that the response rate for a website physical activity program can be as high as 79% when recruiting via telephone [25]. Consequently, in future studies, personal (face-to-face or telephone) contact should be explicitly demanded when recruiting for Web-based interventions. Despite low initial response rates via general practice, most participants believed that GPs promoting physical activity was useful.

The number of participants actually visiting the website and requesting the computer-tailored step advice was reasonably high (nearly 70%) when compared with other studies. Mailing participants a personal login and password seems an effective strategy to invite them to visit the website. Figures were lower in previous Belgian [34] and Australian [25] research, showing that only 53.1% and 46%, respectively, visited the website. It is interesting to note that participants already meeting the 10,000 steps guideline were less likely to request the computer-tailored step advice; this might be due to the 7-day baseline pedometer measurement, suggesting to those participants that there was no real need to visit a website to help them increase their steps. Those who did not request the advice indicated lack of time as the main reason for not doing so. Therefore, in the future, strategies should be developed to encourage those participants to overcome this barrier. Current and future technology might reduce this problem, as Internet access, also due to mobile devices, continues to increase and is now available at several public places such as Wi-Fi hot spots.

Some limitations need to be mentioned. First, as mentioned above, the small sample, which was mainly female, highly educated, employed, and in good health, is the main weakness of the present study. As such, the generalizability of the present findings is limited. Second, the lack of information on how GPs spread the invitation letters confines our understanding of the low initial response rate. In addition, we do not know what the dissemination strategy of the GPs was: did they hand out the invitation letters to their first 50 patients or only to those who were most in need of a physical activity intervention? We also do not know how motivating GPs were during recruitment. The fact that we collected no data on the recruitment process from GPs is a limitation in terms of understanding the poor retention rates. A final weakness is the use of self-reports, which may be subject to recall [16] and social desirability [50] biases. However, we used more objective, pedometer-based step counts to assess physical activity, which is a strong point of the study. The major strength here is the innovative approach of developing pedometer-based and computer-tailored physical activity step advice, which was never used before in an adult population. However, to truly test the impact of this newly developed intervention tool, the present preliminary results need to be confirmed in a larger, sufficiently powered trial applying more successful recruitment methods (eg, telephone contact or online advertising after personally handing out an invitation letter) and in other specific (patient) populations (eg, patients with type 2 diabetes or with cardiovascular disease).

Existing interventions promoting step count increases could benefit from an additional computer-tailored component. For example, community-based interventions guided by socioecological models of health behavior, such as 10,000 Steps Rockhampton, Canada on the Move, and 10,000 Steps Ghent, focus primarily on social systems, policy, and organizations [51]. Individual approaches are less commonly realized [52]. By adding or integrating a computer-tailored component to the community-based interventions, this shortcoming could be overcome, which might increase the effectiveness of these types of programs. In addition, this new tool could be valuable as a stand-alone intervention or as a part of a more comprehensive program in specific groups, such as older people, or patients with type 2 diabetes or other chronic illnesses, after being evaluated in these populations.

To conclude, we describe the development of a new pedometer-based, computer-tailored step advice intervention, which was disseminated through GPs. Despite the poor results of the recruitment method, participants evaluated the dissemination through general practice positively and found it useful for GPs to promote physical activity. A substantial number of participants requested the computer-tailored step advice and rated the acceptability of the tool very well. However, the tailored condition showed no superior effects on self-reported and pedometer-based physical activity, BMI, or time spent sitting, compared with the standard condition. More research is needed to enhance our knowledge of the best dissemination channel and the effectiveness of this tool in larger trials.

## Multimedia Appendix 1

Examples of screenshots of the web-based computer-tailored and pedometer-based physical activity advice.

## Multimedia Appendix 2

CONSORT-EHealth V1.6 Checklist [53].

## Abbreviations

BMI: body mass index
GP: general practitioner
IPAQ: International Physical Activity Questionnaire
