# Supplementary material for: Web-Based, Computer-Tailored, Pedometer-Based Physical Activity Advice: Development, Dissemination Through General Practice, Acceptability, and Preliminary Efficacy in a Randomized Controlled Trial
Source: J Med Internet Res. 2012 Apr 24;14(2):e53. doi: 10.2196/jmir.1959 (PMC3376513; doi:10.2196/jmir.1959)
Supplement: Supplementary file 1 [file jmir_v14i2e53_app1.pptx]

## Slide 1
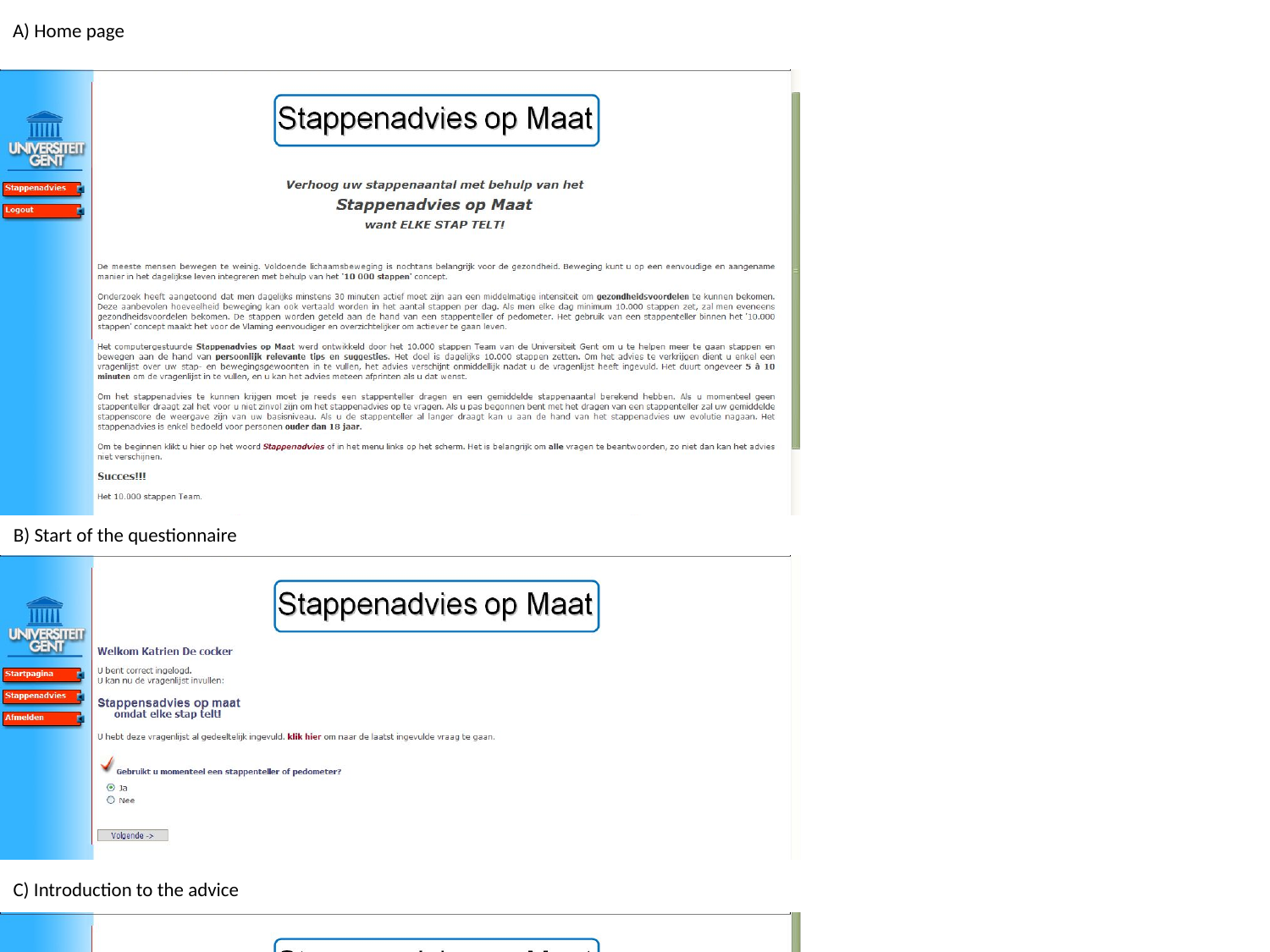

A) Home page
B) Start of the questionnaire
C) Introduction to the advice
D) Schedule to reach a personal step goal
E) Tips and suggestions
