# Supplementary material for: Web-Based, Computer-Tailored, Pedometer-Based Physical Activity Advice: Development, Dissemination Through General Practice, Acceptability, and Preliminary Efficacy in a Randomized Controlled Trial
Source: J Med Internet Res. 2012 Apr 24;14(2):e53. doi: 10.2196/jmir.1959 (PMC3376513; doi:10.2196/jmir.1959)
Supplement: Supplementary file 2 [file jmir_v14i2e53_app2.pdf]

# CONSORT-EHEALTH Checklist V1.6 Report

**Manuscript Number** 1959

**Date completed** 8/25/2011 14:50:29

**by** De Cocker

Web-based computer-tailored and pedometer-based physical activity advice: development, dissemination through general practice, acceptability and preliminary efficacy in a randomized controlled trial.

## TITLE

**1a-i) Identify the mode of delivery in the title**

Yes, see first part of the title: Web-based computer-tailored and pedometer-based physical activity advice...

**1a-ii) Non-web-based components or important co-interventions in title**

Not relevant: there were no non-weg-based compnentes or co-intervetnions.

**1a-iii) Primary condition or target group in the title**

Yes, the title explains that people are recuited through general practice: Web-based computer-tailored and pedometer-based physical activity advice: development, dissemination through general practice, acceptability and preliminary efficacy in a randomized controlled trial.

## ABSTRACT

**1b-i) Key features/functionalities/components of the intervention and comparator in the METHODS section of the ABSTRACT**

Yes, see abstract: Ninety-two participants were recruited through general practitioners (GP) and randomized in a ‘standard condition’ (receiving pedometer only intervention, n=47) and ‘tailored condition’ (receiving pedometer + automated computer-tailored step advice, n=45).

**1b-ii) Level of human involvement in the METHODS section of the ABSTRACT**

Yes, see abstract: ...and ‘tailored condition’ (receiving pedometer + automated computer-tailored step advice...

**1b-iii) Open vs. closed, web-based (self-assessment) vs. face-to-face assessments in the METHODS section of the ABSTRACT**

Yes, see abstract: Ninety-two participants were recruited through general practitioners (GP) and randomized in a ‘standard condition’ (receiving pedometer only intervention, n=47) and ‘tailored condition’ (receiving pedometer + automated computer-tailored step advice, n=45). Step counts, self-reported data obtained via telephone interview on physical activity, sitting time and body mass index (BMI) were assessed at baseline and post-intervention.

**1b-iv) RESULTS section in abstract must contain use data**

Yes, see abstract: Recruitment through GPs was poor (n=107, initial response rate = 6.2%), however, the majority of participants (72.5%) believed it is useful to promote physical activity through general practice. Nearly 70% of the tailored condition requested the computer-tailored step advice and the majority found it understandable (100%), credible (94.4%), relevant (83.3%), not too long (72.2%), instructive (72.2%) and encouraging to increase steps (66.7%).

**1b-v) CONCLUSIONS/DISCUSSION in abstract for negative trials**

Yes, see abstract: The majority of participants in the tailored condition accepted the step advice and indicated it was useful. However, in this selected sample of adults, the tailored condition did not show superior effects compared with the standard condition.

## INTRODUCTION

**2a-i) Problem and the type of system/solution**

yes, see page 3-4: The existing computer-tailored physical activity programs might however also have weaknesses. As the diagnostic assessment is mostly done by questionnaires [9], the self-reported data may suffer from recall biases [16]. Consequently, one could wonder whether the use of more objective outcome measures [11] would be a more appropriate way of assessing baseline physical activity levels in the process of providing computer-tailored feedback. The most commonly used, more objective way of assessing physical activity is measuring step counts through the use of step counters or pedometers [17]. Pedometers have become popular monitoring tools for physical activity in large free-living populations [17]. However, to our knowledge, few computer-tailored interventions in adults have provided feedback based on pedometer use [18,19]. Nevertheless, a number of potential benefits are associated with integrating pedometers in computer-tailored physical activity feedback. Firstly, the accuracy of the assessment of the targeted behaviour will increase, as it would be based on more objectively measured step counts. Secondly, the computer-tailored advice could specifically target step count increases through pedometer use, as such, the pedometer would not only be used as assessment instrument but also as an intervention tool. Due to the self-monitoring aspect and the ongoing feedback provided by the pedometer, resulting in increased awareness and motivation, the device can be used as behaviour modification tool. In addition, goal-setting, a behavioural change strategy used in many computer-tailored programs [9,10,20,21], can easily be facilitated through pedometer use [22]. Pedometer-based behavioural modification programs have already shown positive effects [23,24], however providing continuous face-to-face feedback is time-consuming and expensive. Offering an additional online computer-tailored tool in pedometer-based interventions might thus be beneficial.
Another weakness of online physical activity programs is reaching the targeted population. Recruiting individuals to visit website programs on health behaviour change appears to be rather difficult. For example, Australian research, conducted in a worksite sample, showed that only 46% of participants that agreed to take part in a website-delivered physical activity intervention actually visited the website [25]. A computer-tailored program wherein computers and printers were installed in waiting rooms of general practices in Rhode Island also showed low rates (0-12%) of use [26]. However, a Belgian study revealed that a short face-to-face contact when handing out flyers increased recruitment rates (46%) when compared with recruiting via flyers only (6%) [20]. As such, a possible promising dissemination channel of online physical activity interventions is the visit to primary health care [26], as general practitioners (GPs) have personal face-to-face contact with their patients, but often lack the time or the skills to provide counseling on health behaviours themselves [27,28]. In addition, GPs are perceived as a highly credible source of influence concerning health aspects [29].

**2a-ii) Scientific background, rationale: What is known about the (type of) system**

Yes, see page 3-4: The existing computer-tailored physical activity programs might however also have weaknesses. As the diagnostic assessment is mostly done by questionnaires [9], the self-reported data may suffer from recall biases [16]. Consequently, one could wonder whether the use of more objective outcome measures [11] would be a more appropriate way of assessing baseline physical activity levels in the process of providing computer-tailored feedback. The most commonly used, more objective way of assessing physical activity is measuring step counts through the use of step counters or pedometers [17]. Pedometers have become popular monitoring tools for physical activity in large free-living populations [17]. However, to our knowledge, few computer-tailored interventions in adults have provided feedback based on pedometer use [18,19]. Nevertheless, a number of potential benefits are associated with integrating pedometers in computer-tailored physical activity feedback. Firstly, the accuracy of the assessment of the targeted behaviour will increase, as it would be based on more objectively measured step counts. Secondly, the computer-tailored advice could specifically target step count increases through pedometer use, as such, the pedometer would not only be used as assessment instrument but also as an intervention tool. Due to the self-monitoring aspect and the ongoing feedback provided by the pedometer, resulting in increased awareness and motivation, the device can be used as behaviour modification tool. In addition, goal-setting, a behavioural change strategy used in many computer-tailored programs [9,10,20,21], can easily be facilitated through pedometer use [22]. Pedometer-based behavioural modification programs have already shown positive effects [23,24], however providing continuous face-to-face feedback is time-consuming and expensive. Offering an additional online computer-tailored tool in pedometer-based interventions might thus be beneficial.
Another weakness of online physical activity programs is reaching the targeted population. Recruiting individuals to visit website programs on health behaviour change appears to be rather difficult. For example, Australian research, conducted in a worksite sample, showed that only 46% of participants that agreed to take part in a website-delivered physical activity intervention actually visited the website [25]. A computer-tailored program wherein computers and printers were installed in waiting rooms of general practices in Rhode Island also showed low rates (0-12%) of use [26]. However, a Belgian study revealed that a short face-to-face contact when handing out flyers increased recruitment rates (46%) when compared with recruiting via flyers only (6%) [20]. As such, a possible promising dissemination channel of online physical activity interventions is the visit to primary health care [26], as general practitioners (GPs) have personal face-to-face contact with their patients, but often lack the time or the skills to provide counseling on health behaviours themselves [27,28]. In addition, GPs are perceived as a highly credible source of influence concerning health aspects [29].

## METHODS

**3a) CONSORT**

Yes, see page 4: The objectives of the present paper were to (1) describe the development of an online pedometer-based and computer-tailored physical activity step advice; (2) report on the dissemination of this tool through general practice; (3) report on the perceived acceptability of it in participants recruited through GPs; and (4) evaluate preliminary efficacy of the new intervention in comparison with a standard ‘pedometer only’ intervention.

**3b-i) Bug fixes, Downtimes, Content Changes**

Not relevant: no inmportant changes were made on the intervention or comparator during the study.

**4a-i) Computer / Internet literacy**

Yes, see page 6: ...and presented inclusion criteria i.e. (1) being aged 18-65 years; (2) having Internet access at home or at work; and (3) having a personal e-mail-address.

**4a-ii) Open vs. closed, web-based vs. face-to-face assessments:**

Yes, see pages 6-7: Recruitment of participants was done through GPs, who were asked to personally hand out invitation letters to the 50 first counseling patients eligible for the study.

In order to subscribe, participants were required to e-mail full name, address, telephone number, date of birth, and name of GP to the researchers. Upon receiving subscription information from the participants, they were send an envelope containing a pedometer, a step log for seven days, information on how to use these instruments, and a stamped and addressed envelope for return mailing after three months. Participants were first asked to wear the pedometer for seven consecutive days and to complete the step log in order to assess their baseline step level; they were asked not to increase their step or activity levels from what they usually do in this period. Afterwards, the step log had to be e-mailed to the researchers. After receiving the step log, researchers contacted the participants by telephone to complete the baseline assessment.

During this interview at baseline and post-intervention, participants were asked to report their gender, age, height and weight. The interviewers also obtained information on participants' perceived health (very good; good; moderate; weak; very weak), education (primary education; vocational secondary education; technical secondary education; general secondary education; college, university), employment status (yes; no), computer and Internet use (daily; weekly; monthly; couple of times a year; never), and Internet access at home and at work (yes; no). Furthermore, participants were asked who gave them the invitation letter (GP; others: in case of 'others', participants were asked to specify). Finally, the intention towards physical activity was assessed by asking whether participants planned to increase their steps (yes, within one month; yes, within six months; no intention).

International Physical Activity Questionnaire (IPAQ). To assess physical activity and sitting time, the long interview form of the IPAQ International Physical Activity Questionnaire (IPAQ) was used at baseline and post-intervention.

**4a-iii) Information giving during recruitment**

Yes, see page 6: Recruitment of participants was done through GPs, who were asked to personally hand out invitation letters to the 50 first counseling patients eligible for the study. Exclusion criteria were (1) being physically unable to engage in physical activity; (2) already being highly active or participating in sport activities; and (3) not being Dutch speaking. The letter briefly explained the purpose of the project, namely promoting physical activity in the general population through pedometer use and providing computer-tailored feedback; invited the patient to subscribe in the study; and presented inclusion criteria i.e. (1) being aged 18-65 years; (2) having Internet access at home or at work; and (3) having a personal e-mail-address. In order to subscribe, participants were required to e-mail full name, address, telephone number, date of birth, and name of GP to the researchers.

**4b-i) Report if outcomes were (self-)assessed through online questionnaires**

Yes, see page 7: General Baseline telephone interview. During this interview at baseline and post-intervention, participants were asked to report their gender, age, height and weight. The interviewers also obtained information on participants'...

International Physical Activity Questionnaire (IPAQ). To assess physical activity and sitting time, the long interview form of the IPAQ International Physical Activity Questionnaire (IPAQ) was used at baseline and post-intervention.

Feasibility interview. At post-intervention, feasibility of disseminating through GPs was assessed by asking ...

Acceptability interview. At post-intervention, participants in the tailored condition were asked about...

**4b-ii) Report how institutional affiliations are displayed**

All correspondence and the website had the logo of the Ghent University. This was not reported in the paper as we believe it is not relevant for the results.

**5-i) Mention names, credential, affiliations of the developers, sponsors, and owners**

In an implicit way, yes: we refer to our previous studies on computer-tailoring on which this advice was based, so the information of the affiliation and developers can be found in the reference list.

**5-ii) Describe the history/development process**

Yes, see page 4: Based on previous computer-tailored interventions to increase physical activity [31-34], a computer-tailored step advice was developed. In this web-based intervention participants received personalised feedback on the amount of steps they take on a daily basis, and are also provided with tips and suggestions on how they can take more steps if needed. The general approach, the structure and theoretical background of the new computer-tailored step advice remained the same as in the previous developed computer-tailored interventions, however the focus was changed from increasing physical activity to increasing steps, and as such the 10,000 Steps concept [35,36] was integrated into the present intervention.

**5-iii) Revisions and updating**

Yes, see page 4: Based on previous computer-tailored interventions to increase physical activity [31-34], a computer-tailored step advice was developed. In this web-based intervention participants received personalised feedback on the amount of steps they take on a daily basis, and are also provided with tips and suggestions on how they can take more steps if needed. The general approach, the structure and theoretical background of the new computer-tailored step advice remained the same as in the previous developed computer-tailored interventions, however the focus was changed from increasing physical activity to increasing steps, and as such the 10,000 Steps concept [35,36] was integrated into the present intervention.

**5-iv) Quality assurance methods**

Yes, page 7: Acceptability interview. At post-intervention, participants in the tailored condition were asked about understandability, logic, practical use, and length of the questionnaire prior to receiving the advice. Four questions assessed what participants did with the advice (read it, discussed it with others, saved it, reread it later). The interviewer also asked what the advice indicated about the step level of participants (insufficient; just enough; sufficient), and whether participants were aware of this. Further, participants were asked about the relevance, credibility, understandability, and length of the advice; whether the advice helped them to gain insight in their physical activity pattern; and whether the advice was an encouragement to increase steps. If participants requested the advice more than once, they were asked about the usefulness of receiving the advice twice or more.

**5-v) Ensure replicability by publishing the source code, and/or providing screenshots/screen-capture video, and/or providing flowcharts of the algorithms used**

Yes, some screenshots of the original website (in Dutch) will be provided.

**5-vi) Digital preservation**

No, as this was only a pilot study, the website is not available to the public yet. Consequently, no URL or Internet Archive could be provided.

**5-vii) Access**

Yes, see page 4: Prior to visiting the computer-tailored website, participants' baseline step level had to be determined. In order to receive the computer-tailored step advice, participants had to log onto a website using a confidential username and password, and then complete a questionnaire. This questionnaire assessed participant's demographics, baseline step level and the psychosocial correlates of achieving 10,000 steps/day. As soon as participants had completed all the questions, tailored feedback was provided on the computer screen.

Page 6: Participants in the tailored condition also received a login and password to enter the website that provided the computer-tailored step advice.

**5-viii) Mode of delivery, features/functionalities/components of the intervention and comparator, and the theoretical framework**

Yes, see page 4-5: This feedback was created from a database filled with messages that match any possible combination of answers and is based on the theory of planned behaviour [37] and the transtheoretical model [38]. The theory of planned behaviour was considered by giving feedback about participants' intentions, attitudes, self-efficacy, social support, knowledge, benefits and barriers of physical activity. The stages of changes were considered in two ways. First, the content differed between stages. Precontemplators mainly received general information about the 10,000 Steps concept and about its health benefits. To avoid resistance, the need for behaviour change was not dictated, but only vaguely suggested. Contemplators received the same information, although not so extensively, and it was mentioned that they might benefit from stepping more. In the preparation stage, the emphasis was on increasing steps, combined with specific step and health information. In the action stage, the emphasis was on keeping the steps up and relapse prevention. In the maintenance stage, feedback was reduced to saying that they were doing well and that they should carry on. Second, the way in which the participants were approached also differed between stages. Information for precontemplators was presented in an impersonal way (e.g. people could...), again avoiding resistance. Contemplators were approached in a personal way (e.g. you could…), but not in a decisive way which was used for preparators (e.g. you should…) or a supporting way used for people in the action or maintenance phase (e.g. you do…).

The feedback was organised so that participants first received a general introduction, followed by normative feedback which relates participants' step level to the goal of 10,000 steps/day. Based on baseline step levels, a schedule was provided on how they can reach the goal of 10,000 steps/day over time (participants could choose to increase current steps by 500 or 1000 per week [39]). Progress feedback (positive or negative evolution) was provided when participants requested the advice for a second time or more; it compared the previous step level with the current level. Next, participants received tips on how to increase steps (if needed) during work, household chores, gardening, leisure-time and transport. This further included information on what a 'walking buddy' is, how step guidelines compare with overall physical activity guidelines, how to correctly use a pedometer, what benefits originate from 10,000 steps/day, how to deal with barriers associated with stepping more, how to overcome a low self-efficacy to step more, and how the local environment can provide opportunities to walk. Altogether the feedback, which can be printed, could amount to as much as five or six pages of advice. Table 1 provides some examples of the introduction to the part with tips and suggestions of the advice for the different stages of change.

Table 1: Example of introduction to the tips and suggestion of the advice for the different stages of change.

**5-ix) Describe use parameters**

Yes, see page 5: The feedback was organised so that participants first received a general introduction, followed by normative feedback which relates participants' step level to the goal of 10,000 steps/day. Based on baseline step levels, a schedule was provided on how they can reach the goal of 10,000 steps/day over time (participants could choose to increase current steps by 500 or 1000 per week [39]). Progress feedback (positive or negative evolution) was provided when participants requested the advice for a second time or more; it compared the previous step level with the current level. Next, participants received tips on how to increase steps (if needed) during work, household chores, gardening, leisure-time and transport.

**5-x) Clarify the level of human involvement**

Yes, see page 4: As soon as participants had completed all the questions, tailored feedback was provided on the computer screen. This feedback was created from a database filled with messages that match any possible combination of answers and is based on the theory of planned behaviour [37] and the transtheoretical model [38].

**5-xi) Report any prompts/reminders used**

Yes, see page 6: Participants in the tailored condition also received a login and password to enter the website that provided the computer-tailored step advice. Every month, researchers checked whether participants requested the computer-tailored step advice. If they did, an invitation to receive the computer-tailored step advice for a second or third time was e-mailed in order to receive feedback on their progress. If they did not, a reminder was e-mailed to re-invite participants to request the computer-tailored step advice for the first time.

**5-xii) Describe any co-interventions (incl. training/support)**

Not relevant: there were no co-interventions in the present study.

**6a-i) Online questionnaires: describe if they were validated for online use and apply CHERRIES items to describe how the questionnaires were designed/deployed**

Not relevant, as measures were obtained throuhg telephone interview and not through online questionnaires.

**6a-ii) Describe whether and how "use" (including intensity of use/dosage) was defined/measured/monitored**

Yes, see page 6: Every month, researchers checked whether participants requested the computer-tailored step advice.

**6a-iii) Describe whether, how, and when qualitative feedback from participants was obtained**

Yes, see page 7: Acceptability interview. At post-intervention, participants in the tailored condition were asked about understandability, logic, practical use, and length of the questionnaire prior to receiving the advice. Four questions assessed what participants did with the advice (read it, discussed it with others, saved it, reread it later). The interviewer also asked what the advice indicated about the step level of participants (insufficient; just enough; sufficient), and whether participants were aware of this. Further, participants were asked about the relevance, credibility, understandability, and length of the advice; whether the advice helped them to gain insight in their physical activity pattern; and whether the advice was an encouragement to increase steps. If participants requested the advice more than once, they were asked about the usefulness of receiving the advice twice or more.

**7a-i) Describe whether and how expected attrition was taken into account when calculating the sample size**

No sample size calculations were conducted a priori. We provided the general practitioners with 1900 invitation letters. The response rate was disappointing low (6.2%) so power calculation were done post hoc, see page 13: A priori power analysis indicated that 23 individuals in each intervention condition (total n=46) should have participated to achieve sufficient power. This was the case for most self-reported data, but unfortunately only 20 did actually provide objective step count data on both baseline and post-intervention measurements.

**7b) CONSORT**

Not applicable/relevant for the present study.

**8a) CONSORT**

Yes, see page 6: participants were randomly assigned to: (1) pedometer intervention only ('standard' condition); or (2) pedometer intervention supplemented with computer-tailored step advice ('tailored' condition).

**8b) CONSORT**

Yes, see page 6: participants were randomly assigned to: (1) pedometer intervention only ('standard' condition); or (2) pedometer intervention supplemented with computer-tailored step advice ('tailored' condition).

**9) CONSORT**

Yes, see page 6: participants were randomly assigned to: (1) pedometer intervention only ('standard' condition); or (2) pedometer intervention supplemented with computer-tailored step advice ('tailored' condition).

**10) CONSORT**

Yes, see page 6: participants were randomly assigned to: (1) pedometer intervention only ('standard' condition); or (2) pedometer intervention supplemented with computer-tailored step advice ('tailored' condition).

**11a-i) Specify who was blinded, and who wasn't**

It was not possible to blind the participants as different interviews were used for the different conditions.

**11a-ii) Discuss e.g., whether participants knew which intervention was the "intervention of interest" and which one was the "comparator"**

Participants were not aware of the "comparator" as this was not a truly control groups. This was addressed in the paper, see page 13: the present standard condition was not a truly non-intervention group

**11b) CONSORT**

Yes, see page 6: After this interview, participants were randomly assigned to: (1) pedometer intervention only ('standard' condition); or (2) pedometer intervention supplemented with computer-tailored step advice ('tailored' condition). Participants in both conditions were mailed generic paper booklets with information on how to increase steps [35,36]. Participants in the tailored condition also received a login and password to enter the website that provided the computer-tailored step advice.

**12a) CONSORT**

Yes, see page 8: The time and intervention effects on body mass index (BMI), self-reported and pedometer-based physical activity, and sitting time were examined using repeated measures ANOVAs with "condition" as between-subjects factor and "time" as within-factor.

**12a-i) Imputation techniques to deal with attrition / missing values**

Yes, see page 8: These analyses were executed using both a retained sample analyses (only participants who completed post-intervention assessments) and an intent-to-treat analysis (assuming baseline values at post-intervention for drop-out participants). As no differences were found between both types of analyses, only results on the retained sample analyses are reported.

**12b) CONSORT**

Yes, see page 8: The time and intervention effects on body mass index (BMI), self-reported and pedometer-based physical activity, and sitting time were examined using repeated measures ANOVAs

See page 12: In the tailored condition only, no intervention or time effects were found for the group who did request the advice and those who did not (data not shown).

RESULTS

**13a) CONSORT**

Yes, see figure 1 (page 10: flow chart)

**13b) CONSORT**

Yes, see figure 1 (page 10: flow chart) +

see page 9: In total, 23 participants dropped out, seven because of health problems, three because of a lack of time and one went abroad. The other 12 drop-out participants could not be reached at post-intervention, so the reason for drop-out in unknown.

See page 9: From the 1900 available invitation letters (50 per GP, 38 GPs), 1737 letters were handed out to patients. A total of 107 individuals subscribed to participate (response rate = 6.2%), however one participant did not meet inclusion criteria and seven eventually withdrew to participate, due to family- or work-related reasons; leaving 99 participants at baseline (see Figure 1). The baseline interview (completed by 92 participants) revealed that 89 participants had received the invitation letter from the GP, one person found it in the waiting room, one received it from his wife and one received it from a parent.

**13b-i) Attrition diagram**

Not applicible/relevant, as the website was no longer available after the intervention.

**14a) CONSORT**

Yes, see page 6: The study was conducted between January and August 2010.

**14a-i) Indicate if critical "secular events" fell into the study period**

Not applicable/relevant for this study.

**14b) CONSORT**

Not applicable/relevant for this study.

**15) CONSORT**

Yes, see table 2 page 8-9.

**15-i) Report demographics associated with digital divide issues**

Yes, the following variables were shown in table 2: Demographic variables: Age, gender, BMI, education, employment and health.

Use PC/Internet and Internet access: Daily PC-use (%) Daily Internet-use (%), Internet access at home (%), Internet access at work (%)

**16-i) Report multiple "denominators" and provide definitions**

Yes, see abstract (page 2), table 2 (page 8-9), figure 1 (page 10: flow chart), table 3 (page 11), table 4 (page 12) and associated text.

**16-ii) Primary analysis should be intent-to-treat**

Yes, see page 8: These analyses were executed using both a retained sample analyses (only participants who completed post-intervention assessments) and an intent-to-treat analysis (assuming baseline values at post-intervention for drop-out participants). As no differences were found between both types of analyses, only results on the retained sample analyses are reported.

**17a) CONSORT**

yes, see table 4 (page 12):
Variable/condition
n
Baseline (mean ± SD)
Post-intervention (mean ± SD)
Change (95% CI)
Ftimexcondition P
Ftime  P

**17a-i) Presentation of process outcomes such as metrics of use and intensity of use**

Yes, see page 11-12: Most frequently mentioned reason for not requesting the advice was lack of time; one person had computer problems; and one believed that he didn't need the advice. Of those who did request the advice, all found the questions prior to receiving the advice understandable (100%), the majority had no problems answering them (95.7%), found them logically build up (95%), and didn't find the questionnaire too long (73.7%). After receiving the advice, almost everyone read it (95.2%) and the majority saved it (60.0%). Less participants discussed it with others (42.1%), printed it (38.1%) and reread it later (35.0%). Of those who could remember the feedback on their step level, almost half (47.4%) got the advice they were insufficiently active. The majority (68.4%) had expected the feedback they got. Of those requesting the advice more than once (n=7), everyone found it useful to be able to receive the advice several times.

**17b) CONSORT**

Not appicable/relevant

**18) CONSORT**

Yes see page 12: In the tailored condition only, no intervention or time effects were found for the group who did request the advice and those who did not (data not shown).

**18-i) Subgroup analysis of comparing only users**

Yes, see page 12: In the tailored condition only, no intervention or time effects were found for the group who did request the advice and those who did not (data not shown).

**19) CONSORT**

Not applicable/relevant for this study as no harms or unintented effects were found.

**19-i) Include privacy breaches, technical problems**

Not applicable/relevant as no privacy breaches or technical problems occured.

**19-ii) Include qualitative feedback from participants or observations from staff/researchers**

Those who dropped-out did nog use the application as intended. This was addressed on page 9: In total, 23 participants dropped out, seven because of health problems, three because of a lack of time and one went abroad.

## DISCUSSION

**20-i) Typical limitations in ehealth trials**

Yes, see page 14: Some limitations need to be mentioned. Firstly, as mentioned above, the small sample which was mainly female, highly educated, employed, and in good health, is the main weakness of the present study. As such the generalizability of the present findings is limited. Second, the lack of information on how GPs spread the invitation letters confines our understanding of the low initial response rate. In addition, it is not known what the dissemination strategy of the GPs was: whether or not they handed out the invitation letters to the 50 first patients, or only to those who were most in need of a physical activity intervention? It is also unknown how motivating GPs were during recruitment? The fact that no data on the recruitment process was collected from GPs is the main limitation in terms of understanding the poor retention rates. A final weakness is the use of self-reports that may suffer from recall [16] and social desirability [50] biases. However more objective pedometer-based step counts were used to assess physical activity, which is a strong point of the study. The major strength here is the innovative approach of developing pedometer-based and computer-tailored physical activity step advice, which was never used before in an adult population.

**21-i) Generalizability to other populations**

Yes, see page 14: Some limitations need to be mentioned. Firstly, as mentioned above, the small sample which was mainly female, highly educated, employed, and in good health, is the main weakness of the present study. As such the generalizability of the present findings is limited.

**21-ii) Discuss if there were elements in the RCT that would be different in a routine application setting**

Not applicable/relevant for the present study, as this intervention tool was web-based and automated without components needing personal contact.

**22-i) Restate study questions and summarize the answers suggested by the data, starting with primary outcomes and process outcomes (use)**

Yes, see page 12-13: This study presented a new pedometer-based and computer-tailored step advice and examined the feasibility of disseminating this tool through general practice, the acceptability and the preliminary efficacy of it in adults. Overall, participants accepted the computer-tailored step advice well.
Despite this positive evaluation of the computer-tailored step advice, the tool did not result in significant effects on behaviour or BMI, compared with the condition not receiving the advice.

**22-ii) Highlight unanswered new questions, suggest future research**

Yes, see page 14: However, to truly test the impact of this newly developed intervention tool, the present preliminary results need to be confirmed in a larger sufficiently powered trial applying more successful recruitment methods (e.g. telephone contact after personally handing out an invitation letter), and in other specific (patient) populations (e.g. type 2 diabetes patients, patients with cardiovascular disease).

## Other information

**23) CONSORT**

This paper includes several parts (development of step advice; dissemination results; acceptability results; and efficacy results) of which the small trial was one. This trial was not registered as it was originally set up as a pilot study, in order to obtain preliminary data prior to executing large intensive clinical trials.

**24) CONSORT**

Not applicable as all relevant information about the study is in the paper.

**25) CONSORT**

Yes, see page 15:
De Cocker K was supported by the Research Foundation Flanders (FWO) (post-doctoral research fellowship: FWO11/PDO/097).
Vandelanotte C was supported by a National Health and Medical Research Council of Australia (#519778) and National Heart Foundation of Australia (#PH 07B 3303) post-doctoral research fellowship.

**X26-i) Comment on ethics committee approval**

Yes, see page 6: Participants completed informed consent forms and the study protocols were approved by the Ethical Committee of the Ghent University.

**x26-ii) Outline informed consent procedures**

Participants completed informed consent forms and the study protocols were approved by the Ethical Committee of the Ghent University.

**X26-iii) Safety and security procedures**

Yes, see page 6: Exclusion criteria were (1) being physically unable to engage in physical activity;...

**X27-i) State the relation of the study team towards the system being evaluated**

Yes, see page 15: No financial or other conflicts of interest are declared. The authors are identical with the developers of the intervention.
